# A comparative multicentre study evaluating gluteal turnover flap for wound closure after abdominoperineal resection for rectal cancer

**DOI:** 10.1007/s10151-021-02496-7

**Published:** 2021-07-14

**Authors:** S. Sharabiany, J. J. W. van Dam, S. Sparenberg, R. D. Blok, B. Singh, S. Chaudhri, F. Runau, A. A. W. van Geloven, A. W. H. van de Ven, O. Lapid, R. Hompes, P. J. Tanis, G. D. Musters

**Affiliations:** 1 Department of Surgery, Amsterdam UMC, University of Amsterdam, Post-box 22660, 1100 DD Amsterdam, The Netherlands; 2 Department of Surgery, Leicester University Hospital, Leicester, UK; 3 Department of Surgery, Tergooi Hospital, Hilversum, The Netherlands; 4 Department of Surgery, Flevo Hospital, Almere, The Netherlands

**Keywords:** Perineal wound healing, Abdominoperineal resection, Rectal cancer, Wound closure

## Abstract

**Background:**

The aim of this study was to compare perineal wound healing between gluteal turnover flap and primary closure in patients undergoing abdominoperineal resection (APR) for rectal cancer.

**Methods:**

Patients who underwent APR for primary or recurrent rectal cancer with gluteal turnover flap in two university hospitals (2016–2021) were compared to a multicentre cohort of primary closure (2000–2017). The primary endpoint was uncomplicated perineal wound healing within 30 days. Secondary endpoints were long-term wound healing, related re-interventions, and perineal herniation. The perineal hernia rate was assessed using Kaplan Meier analysis.

**Results:**

Twenty–five patients had a gluteal turnover flap and 194 had primary closure. The uncomplicated perineal wound-healing rate within 30 days was 68% (17/25) after gluteal turnover flap versus 64% (124/194) after primary closure, OR 2.246; 95% CI 0.734–6.876; *p* = 0.156 in multivariable analysis. No major wound complications requiring surgical re-intervention occurred after flap closure. Eighteen patients with gluteal turnover flap completed 12-month follow-up, and none of them had chronic perineal sinus, compared to 6% (11/173) after primary closure (*p* = 0.604). The symptomatic 18-month perineal hernia rate after flap closure was 0%, compared to 9% after primary closure (*p* = 0.184).

**Conclusions:**

The uncomplicated perineal wound-healing rate after the gluteal turnover flap and primary closure after APR is similar, and no chronic perineal sinus or perineal hernia occurred after flap closure. Future studies have to confirm potential benefits of the gluteal turnover flap.

## Introduction

Abdominoperineal resection (APR) for rectal cancer is associated with a high rate of perineal morbidity. Perineal wound complications have been reported to occur in up to 35% of patients within 30 days, and up to 10% have an unhealed perineal wound at 1 year [1, 2]. This high rate of perineal wound complications is most likely due to the large perineal dead space that is created. In addition, the use of neo-adjuvant radiotherapy is known to negatively affect perineal wound healing [3].

In an attempt to manage this problem, several perineal closure techniques have been applied over the past decades. The perineal wound can be closed primarily, with a biological mesh, or by different myo- and fasciocutaneous flaps. Currently, there is no consensus on which method to use. Biological mesh closure has been compared to primary closure in the BIOPEX-study [1]. This randomised multicentre trial showed no significant difference in perineal wound complications at 30 days (37% after biological mesh closure versus 34% after primary closure) [1]. The recently published study of long-term results showed a significantly lower 5-year perineal hernia rate after biological mesh closure (6% vs. 28%) [4].

The absence of any impact on perineal wound healing in the BIOPEX-study was likely related to the remaining dead space at the level of the excised sphincter complex. By filling this space with well-vascularized tissue, accumulation of contaminated fluid with abscess formation might be prevented, besides tensionless closure. Myocutaneous flaps seem to be more effective based on this principle [5]. However, drawbacks of these flaps are donor-site morbidity, the complexity of the reconstructive procedures with the need for a plastic surgeon, and the substantially increased operative time.

The gluteal turnover flap is a small subcutaneous transposition flap, ideally designed for routine closure after APR, and consisting of adjacent skin and subcutaneous tissue of one of the buttocks with a maximum width of 2.5 cm. There is no need for isolating individual perforating arteries. The flap is hinged into the resected space and the de-epithelialized dermis is stitched to the contralateral levator remnant. The subcutaneous fat and perineal skin is closed in layers over the flap in the midline, thereby avoiding an additional scar.

A pilot study by our group demonstrated the feasibility and safety of the gluteal turnover flap in 10 patients with a total follow-up of 1 month [6]. Until the start of the randomised BIOPEX-2 study (NCT04004650), an additional number of patients underwent a gluteal turnover flap in two of the participating centres [7]. The aim of this study was to evaluate the extended experience with gluteal turnover flap closure following APR for rectal cancer, and to compare perineal wound healing and hernia formation after longer follow-up with a control group of primary closure.

## Materials and methods

### Patients and study design

All patients who underwent APR for primary or recurrent rectal cancer with gluteal turnover flap in two academic centres were included. The gluteal turnover flap was introduced in 2016 at the Amsterdam University Medical Centre (AMC) and in 2019 at Leicester University Hospital. All consecutive patients underwent this closure technique, provided that there was no indication for extended resection of the perineal skin or ischioanal fat. Four patients from the previously published pilot study who were treated at the AMC were also included in the present cohort [6]. The control group consisted of an existing cohort of patients with rectal cancer who underwent APR with primary closure in three hospitals in the Netherlands: AMC (2000–2017), Tergooi Hospital (2000–2017), and Flevo Hospital (2010–2017).

Exclusion criteria for both the flap and control group were APR for other underlying disease (i.e., anal cancer or inflammatory bowel disease), intersphincteric APR, total pelvic exenteration, sacral resection above level S4/S5, and biological mesh or muscle flap closure of the pelvic floor.

Data extraction from patient records included baseline characteristics, operative details, perineal wound complications, and related re-interventions and re-admissions. Outcomes after flap closure were evaluated until maximum follow-up for each individual patient.

The Institutional Review Board of the AMC waived the need for informed consent for the primary closure group. Approval was needed and given by the medical ethics committees of the AMC and Leicester University Hospital for the gluteal turnover flap group and informed consents were collected from all patients.

### Outcome measures

The primary endpoint was the percentage of patients with an uncomplicated perineal wound healing within 30 days.

Secondary endpoints included the uncomplicated perineal wound healing rate within 1 year, the perineal wound complication rate at 6 and 12 months (patients with a follow-up shorter than 6 and 12 months were excluded from analysis), the incidence of symptomatic perineal herniation (based on clinical signs), and the related re-interventions and re-admissions.

The Clavien–Dindo Score was used to evaluate all perineal wound complications and related re-interventions within 30 days following APR.

### Technique

The gluteal turnover flap was created with the patient in either prone or lithotomy position. First, an island of adjacent skin of the left or right buttock side was marked by a half-moon shape with a maximum width of 2.5 cm. Second, the skin-island was de-epithelialized and a flap was created by dissecting the subcutaneous fat towards the gluteal muscle at an angle of approximately 45 degrees. The perforating gluteal branches were not identified with Doppler ultrasound and were not separately dissected, as these are abundantly present at this level. Thereafter, the subcutaneous gluteal flap was turned inwards, and the de-epithelialised dermis was fixed to the contralateral pelvic floor remnant with 2.0 Vicryl sutures. Subsequently, midline layered closure of the perineum was performed with a vacuum drain being placed between the gluteal turnover flap and the subcutaneous fat. Postoperatively, patients did not have to be on a pressure relief mattress and were allowed to sit and walk from day 1 post-operative. Pictures of the procedure as well as a video vignette have previously been published [6, 8].

### Statistical analysis

Categorical data were reported as proportions and compared using Fisher's exact test. Numerical data were reported according to distribution in means with standard deviation (SD) or medians with interquartile range (IQR) and compared using the *t* test or Mann–Whitney *U* test, respectively. The primary outcome (i.e., uncomplicated perineal wound healing within 30 days) was compared between the two study groups using a two-sided Fisher’s exact test. Any binary secondary outcome (e.g., perineal hernia rate, re-intervention rate, etc.) was analysed in the same way as the primary outcome. A multivariable logistic regression analysis was performed to determine the effect of perineal closure method on perineal wound healing after correction for potential confounders. The following potential confounders were selected for the primary outcome (i.e., uncomplicated perineal wound healing within 30 days): type of APR, neo-adjuvant treatment, omentoplasty, abdominal approach, multivisceral resection, and APR indication. In addition, American Society of Anesthesiologists (ASA) classification and vascular disease were added for the uncomplicated perineal wound healing rate within 1 year. Results are presented as odds ratio (OR) and 95% confidence Interval (95% CI). Covariates were selected based on pre-existing knowledge and significant differences in baseline characteristics between groups. The maximum number of covariates was dependent on the number of events the data allowed to correct for. To determine potential predictors for increased operative time, a linear regression analysis was performed with ASA classification, neo-adjuvant treatment, APR type, vascular disease, omentoplasty, multivisceral resection, abdominal approach, and gluteal turnover flap. Results are presented as beta (*B*) in minutes and 95% CI. The survival probability and incidence of perineal hernia were determined by Kaplan–Meier analyses and compared using the log-rank test. The statistical significance level was set at a *p* value of < 0.05. Statistical analysis was performed using SPSS software for Windows version 26 (IBM Corp, Armonk, NY, USA).

## Results

### Patient characteristics

Between June 2016 and February 2021, a total of 41 patients underwent APR with gluteal turnover flap closure. After exclusion of APR for other underlying disease (i.e., anal cancer or inflammatory bowel disease; *n* = 15), and total pelvic exenteration (*n* = 1), a total of 25 patients were included in the gluteal turnover flap group. In the control group, a total of 239 patients who underwent APR with primary perineal wound closure between February 2000 and April 2018 were potentially eligible. After exclusion of patients with missing operative records (*n* = 8) and patients who underwent an intersphincteric APR (*n* = 37), a total of 194 patients were included in the primary perineal closure group.

Mean age of the whole group was 67 years, 67% (146/219) of the patients were male, and 77% (169/219) received neo-adjuvant treatment for rectal cancer.

There were significant baseline differences between the groups, with more ASA III patients (32% (8/25) vs. 18% (34/189); *p* = 0.003) and more recurrent rectal cancer (20% (5/25) vs. 1% (1/194); *p* < 0.001) in the flap group (Table 1).

**Table 1:** Baseline characteristics

	Gluteal turnover flap (*n* = 25)	Primary closure (*n* = 194)	*p* value
Sex			
Male	1525 (60)	131/194 (68)	0.452
Age			
Years (mean ± SD)	62 ± 10	65 ± 13	0.142
Body mass index			
Kg/m^2^ (mean ± SD)	29 ± 6	27 ± 4	0.111
ASA classification			
ASA I	0/25 (0)	51/189 (27)	0.003
ASA II	17/25 (68)	103/189 (55)	
ASA III	8/25 (32)	34/189 (18)	
ASA IV	0/25 (0)	1/189 (1)	
Smoking status			
Active	5/25 (20)	23/148 (16)	0.025
Former	1/25 (4)	44/148 (30)	
Never	19/25 (76)	81/148 (55)	
Comorbidities			
Diabetes mellitus	5/25 (20)	29/192 (15)	0.056
Vascular disease	12/25 (48)	34/192 (18)	
Prior abdominal surgery			
Total	11/25 (44)	84/193 (44)	0.964
Prior pelvic surgery			
Total	9/25 (36)	39/193 (20)	0.073
Hysterectomy	2/9 (22)	7/63 (11)	
APR indication			
Primary rectal cancer	20/25 (80)	193/194 (99)	< 0.001
Recurrent rectal cancer	5/25 (20)	1/194 (1)	
Neo-adjuvant treatment			
Total	19/25 (76)	150/194 (77)	0.017
Short-course radiotherapy	1/25 (4)	58/194 (30)	
Long-course radiotherapy^a^	18/25 (72)	92/194 (47)	
Adjuvant chemotherapy			
Total	2/25 (12)	22/192 (12)	1.000
Follow-up duration			
Months (median + IQR)	14 (7–25)	39 (21–77)	< 0.001
One-year survival			
%	91	94	0.716

Data are presented as proportions (%), unless otherwise stated

*SD* standard deviation, *ASA* American Society of Anaesthesiologists, *APR* abdominoperineal resection, *IQR* interquartile range

^a^Chemoradiotherapy and long-course radiotherapy alone

Gluteal turnover flap patients had significantly more often an extralevator APR (100% (25/25) vs. 38% (74/194); *p* < 0.001), and less often an omentoplasty (12% (3/25) vs. 40% (77/194); *p* = 0.007). The use of a perineal drain was significantly higher in the gluteal turnover flap group (100% (25/25) vs. 47% (92/194); *p* < 0.001) (Table 2). The operative time of patients in the gluteal turnover flap group was significantly longer (325 min (IQR 266–385) vs. 223 min (IQR 182–290); *p* < 0.001). In linear regression analysis, extralevator APR (B 40.6; 95% CI 14.8–66.4; *p* = 0.002), omentoplasty (B 65.0; 95% CI 41.0–89.0; *p* < 0.001), and the gluteal turnover flap (B 70.6; 95% CI 34.5 – 106.7; *p* =  < 0.001) were found to be significant predictors for an increase in operative time (Table 3). The median length of post-operative hospital stay was similar between both groups (gluteal turnover flap 9 days (IQR 6–12) vs primary closure 9 days (IQR 6–15); *p* = 0.921).

**Table 2:** Operative details

	Gluteal turnover flap (*n* = 25)	Primary closure (*n* = 194)	*p* value
APR type			
Extralevator	25/25 (100)	74/194 (38)	< 0.001
Conventional	0/25 (0)	120/194 (62)	
Abdominal approach			
Open	2/25 (8)	59/194 (30)	< 0.001
Laparoscopic	13/25 (52)	135/194 (70)	
Robotic	10/25 (40)	0/194 (0)	
Conversion^a^	2/23 (9)	5/135 (4)	0.270
Multivisceral resection			
Total^b^	5/25 (20)	30/194 (16)	0.564
Coccyx	2/25 (8)	2/194 (1)	
Uterus	1/10 (10)	2/63 (3)	
Vaginal wall	2/10 (20)	17/63 (27)	
Partial prostate	1/15 (7)	8/131 (6)	
Pelvic sidewall	2/25 (8)	3/194 (2)	
Urinary tract	2/25 (8)	1/194 (1)	
Presacral fascia	0/25 (0)	1/194 (1)	
Vesicula or adnexa	2/25 (8)	5/194 (3)	
Omentoplasty			
Yes	3/25 (12)	77/194 (40)	0.007
Pelvic drain			
Total	22/25 (88)	146/194 (75)	0.156
Perineal drain			
Total	25/25 (100)	92/194 (47)	< 0.001
Operative time			
Minutes (median + IQR)	325 (266–385)	223 (182–290)	< 0.001
Post-operative hospital stay			
Days (median + IQR)	9 (6–12)	9 (6–15)	0.921

Data are presented as proportions (%), unless otherwise stated

*APR* abdominoperineal resection, *IQR* interquartile range

^a^Percentage from laparoscopic and robotic group

^b^Combined number is smaller than the sum of the separate number

**Table 3:** Linear regression model: operative time

	*B* in minutes	95% CI	*p* value
ASA III and IV^a^	18.64	−10.7–47.9.3	0.212
Neo-adjuvant radiotherapy	17.5	−7.3–42.2	0.166
Extralevator APR^b^	40.6	14.8–66.4	0.002
Vascular disease	3.0	−27.2–33.1	0.847
Omentoplasty	65.0	41.0–89.0	< 0.001
Adjacent organ resection	22.0	−6.5–50.5	0.130
Open abdominal approach^c^	−16.7	−41.0–7.6	0.178
Gluteal turnover flap	70.6	34.5–106.7	< 0.001

*B* beta, *CI* confidence interval, *ASA* American Society of Anaesthesiologists classification, *APR* abdominoperineal resection

^a^Compared to ASA I and II

^b^Compared to conventional APR

^c^Compared to laparoscopic and robotic approach

### Perineal wound healing

Within 30 days, the percentage of patients with an uncomplicated perineal wound healing was 68% (17/25) after gluteal turnover flap and 64% (124/194) after primary closure (*p* = 0.688) (Table 4). Multivariable logistic regression analysis did not reveal a significant association between gluteal turnover flap and uncomplicated perineal wound healing within 30 days (OR 2.246; 95% CI 0.734 – 6.876; *p* = 0.156) (Table 5).

**Table 4:** Perineal wound outcomes

	Gluteal turnover flap (*n* = 25)	Primary closure (*n* = 194)	*p* value
Uncomplicated perineal wound healing			
Within 30 days	17/25 (68)	124/194 (64)	0.688
Within 1 year	14/25 (56)	112/194 (58)	0.869
Perineal wound complication			
At 6 months	1/23 (4)	18/180 (10)	0.703
At 12 months	0/18 (0)	11/173 (6)	0.604
Specific perineal wound complications^a^			
Major dehiscence	5/25 (20)	35/194 (18)	
Seroma	3/25 (12)	15/194 (8)	
Superficial wound infection	1/25 (4)	14/194 (7)	
Abscess	3/25 (12)	42/194 (22)	
Fistula	0/25 (0)	11/194 (6)	
Haemorrhage	0/25 (0)	16/194 (8)	
Necrosis	0/25 (0)	4/194 (2)	
Non-surgical wound re-intervention^a^			
Total^b^	6/25 (24)	30/194 (16)	0.263
Bedside	5/25 (20)	24/194 (12)	
Radiological	1/25 (4)	10/194 (5)	
Surgical wound re-intervention^a^			
Total	0/25 (0)	14/194 (7)	0.378
Muscle flap repair	0/25 (0)	2/194 (1)	
Abscess drainage	0/25 (0)	5/194 (3)	
Debridement	0/25 (0)	1/194 (1)	
Perineal bleeding	0/25 (0)	3/194 (2)	
Fistula repair	0/25 (0)	3/194 (2)	
Perineal dehiscence	0/25 (0)	1/194 (1)	
Clavien–Dindo classification^c^			
Grade 0	17/25 (68)	152/194 (78)	0.057
Grade I	7/25 (28)	17/194 (9)	
Grade II	0/25 (0)	1/194 (1)	
Grade IIIa	1/25 (4)	10/194 (5)	
Grade IIIb	0/25 (0)	14/194 (7)	
Perineal VAC placement^a^			
Total	2/25 (8)	14/194 (7)	1.000
On ward	2/25 (8)	13/194 (7)	
Under general anaesthesia	0/25 (0)	1/194 (1)	
Perineal wound-related re-admission^a^			
Total	2/25 (8)	25/194 (13)	0.747
Days (median + IQR)	2 (NA)	13 (5–23)	0.051

Data are presented as proportions (*n* %), unless otherwise stated

*VAC* vacuum assisted closure, *IQR* interquartile range, *NA* not applicable

^a^Until one year

^b^Combined number could be smaller than the sum of the separate numbers

^c^Until 30 days postoperatively

**Table 5:** Multivariable logistic regression analysis

Uncomplicated perineal wound healing within 30 days				Uncomplicated wound healing within 1 year			
	OR	95% CI	*p* value		OR	95% CI	*p* value
Unadjusted				Unadjusted			
Gluteal turnover flap	1.200	0.493–2.921	0.689	Gluteal turnover flap	0.932	0.402–2.157	0.869
Adjusted				Adjusted			
Gluteal turnover flap	2.246	0.734–6.876	0.156	Gluteal turnover flap	1.537	0.527–4.480	0.431
Extralevator APR^a^	0.420	0.209–0.846	0.015	Extralevator APR^a^	0.630	0.317–1.252	0.188
Neo-adjuvant treatment	0.575	0.280–1.180	0.131	Neo-adjuvant treatment	0.670	0.340–1.317	0.246
Omentoplasty	1.176	0.611–2.2264	0.628	Omentoplasty	1.340	0.701–2.562	0.375
Open abdominal approach^b^	0.510	0.261–0.999	0.050	Open abdominal approach^b^	0.646	0.335–1.244	0.191
Multivisceral resection	1.687	0.743–3.830	0.211	Multivisceral resection	1.295	0.587–2.853	0.522
Recurrent rectal cancer^c^	0.303	0.048–1.892	0.201	Recurrent rectal cancer^c^	0.553	0.091–3.346	0.519
				ASA III and IV^d^	0.738	0.328–1.659	0.462
				Vascular disease	0.853	0.379–1.917	0.700

*OR* odds ratio, *CI* confidence interval, *APR* abdominoperineal resection, *ASA* American Society of Anesthesiologists

^a^Compared to conventional APR

^b^Compared to laparoscopic and robotic approach

^c^Compared to primary rectal cancer

^d^Compared ASA I and II

Also within 1 year, the uncomplicated perineal wound healing rate did not significantly differ between both groups [56% (14/25) vs. 58% (112/194); *p* = 0.869], and this did not change in multivariable analysis (OR 1.537; 95% CI 0.527–4.480; *p* = 0.431).

At 6 months, 4% (1/25) of patients with the gluteal turnover flap had a perineal wound complication compared to 10% (18/180) after primary closure (*p* = 0.703).

Eighteen patients (18/25) in the gluteal turnover flap group completed 12-month follow-up, and none of them (0/18) had a chronic perineal sinus compared to 6% (11/173) of patients after primary closure (*p* = 0.604).

An abscess occurred in 12% (3/25) of patients after gluteal turnover flap, compared to 22% (42/194) of patients after primary closure (*p* = 0.261). No perineal fistulas, haemorrhage, or necrosis were observed after flap closure, while these complications occurred in the primary closure group in 6% (11/194), 8% (16/194), and 2% (4/194), respectively (*p* = 0.619, *p* = 0.227, *p* = 1.000).

No surgical re-interventions were required after gluteal turnover flap, compared to 7% (14/194) after primary closure (*p* = 0.378).

All complications experienced by gluteal turnover flap patients were Clavien–Dindo grade 1 or 2, except in the case of 1 patient who had a grade 3b complication: a presacral abscess which was treated with percutaneous drainage.

The perineal wound-related re-admission rate was 8% (2/25) after gluteal turnover flap and 13% (25/194) after primary closure (*p* = 0.747), with a median length of hospital stay of 2 days (IQR not applicable) and 13 days (IQR 5–23), respectively (*p* = 0.051).

### Perineal hernia

**Fig. 1:**
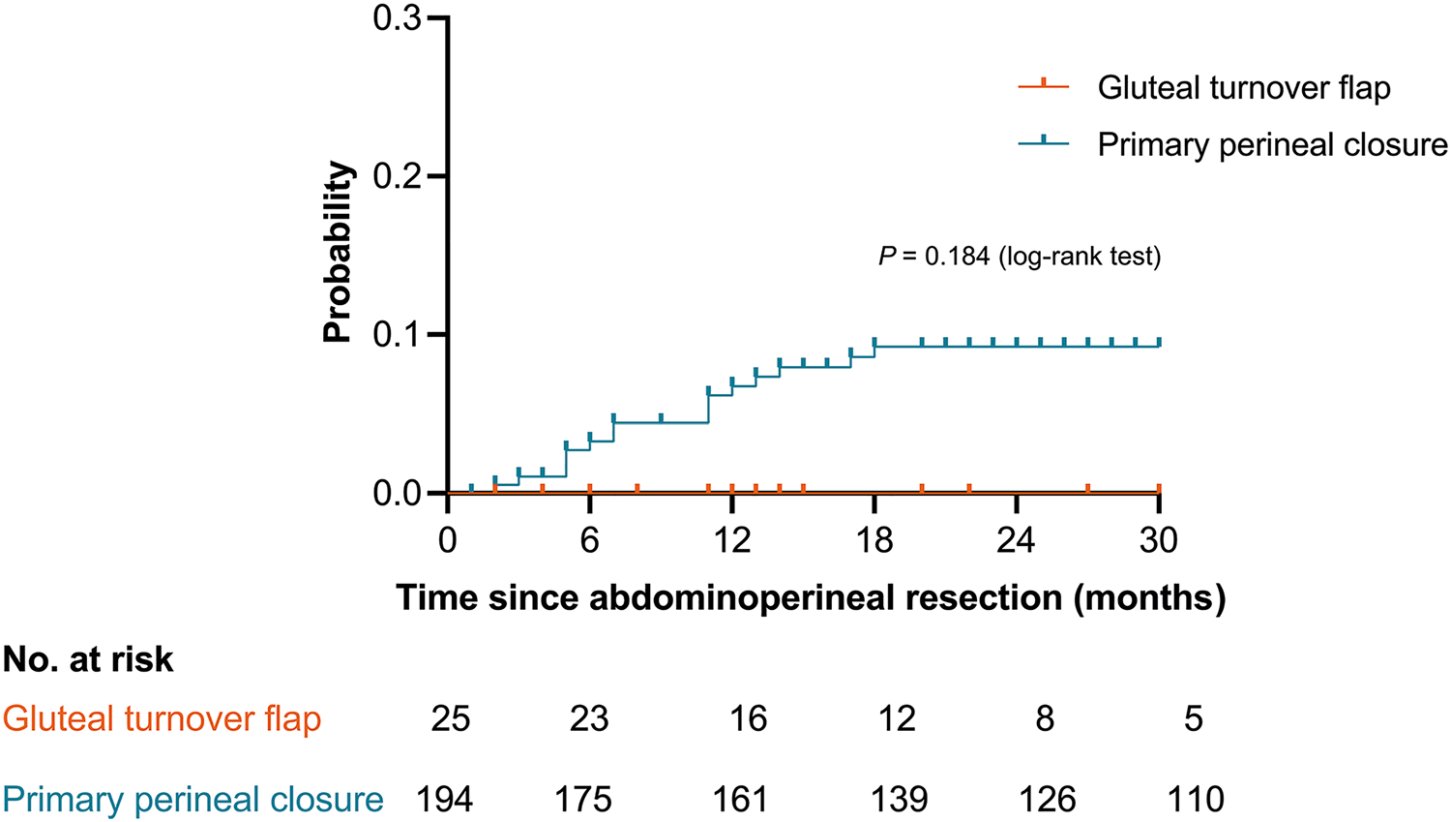
Kaplan–Meier curves of symptomatic perineal hernia after abdominoperineal resection, stratified for method of perineal wound closure: gluteal turnover flap (orange line) and primary perineal closure (blue line). Log rank test was used to test the significance and censored patients were indicated by tick marks. The number of patients at risk is shown at the bottom part of the figure

During follow-up, none of the patients reported clinical signs related to perineal hernia after gluteal turnover flap closure, compared to 24 patients after primary closure (0/25 (0%) vs. 24/194 (12%); *p* = 0.084). The perineal hernias after primary closure were all symptomatic, and 15 patients underwent surgical closure. The 18-month symptomatic perineal hernia rate was 0% after gluteal turnover flap closure and 9% after primary closure (*p* = 0.184; Fig. 1).

## Discussion

This retrospective multicentre cohort study assessed extended experience with a novel perineal wound closure technique after APR for rectal cancer, reporting on outcomes beyond 30 days. The uncomplicated perineal wound-healing rate within 30 days was similar between both groups. None of the patients in the gluteal turnover flap group had a chronic perineal sinus at 12 month, and no flaps were lost or required surgical re-intervention. To date, no symptomatic perineal hernia have developed after gluteal turnover flap. Despite the fact that patient numbers are still small and without significant differences in outcomes, these results are promising and provide additional support for conducting the BIOPEX-2 study [7].

Previous studies assessing perineal closure techniques following APR for rectal cancer have shown similar wound complication rates if compared to primary closure. The use of a biological mesh as an alternative closure method did not result in an improvement of the perineal wound healing, but seems to reduce the risk of perineal herniation [4]. Myocutaneous flaps showed a 17% reduction in wound complications when compared to primary closure in non-randomised comparative studies [5]. The gluteal turnover flap did not show the same reduction in the present study, which likely has several explanations.

First of all, significant differences in baseline characteristics were in favour of the primary closure group. Patients with a gluteal turnover flap had higher ASA scores and significantly more vascular diseases, which are known to be associated with post-operative complications in general and perineal wound complications in particular. [9, 10] The gluteal turnover group also had more patients with recurrent rectal cancer, which contributes to a higher a priori risk of surgical complications and explains the significantly higher rate of prior pelvic surgery in this group. Furthermore, an extralevator APR was performed significantly more often in the gluteal turnover flap patients with more adjacent organ resections, thereby contributing to a larger perineal dead space, and making it more prone to wound complications. For this reason, a multivariable analysis was performed to correct for confounding factors, which revealed a more pronounced difference between the groups in favour of the gluteal turnover flap), although not statistically significant.

Apart from the baseline differences in the present study, comparison with the literature is often complicated by heterogeneous patient populations and several methodological issues. Heterogeneity of the APR cohorts is related to different underlying disease (e.g., might include anal cancer or inflammatory bowel disease as well), degree of radiotherapy, and extent of resections. Methodological issues include retrospective data collection, absence of consensus definitions for perineal wound complications, and high risk of selection and allocation bias. This underlines the need for randomised trials in this field and the use of uniform outcome measures.

It is important to mention that myocutaneous flaps have a necrosis rate of approximately 13% [11, 12]. The absence of flap necrosis, bleeding and surgical re-interventions after gluteal turnover flap closure, illustrates that this subcutaneous flap is a less invasive perineal closure technique, along with not having the other drawbacks of myocutaneous flaps, i.e., donor-site scar, donor-site morbidity, and the need for a plastic surgeon. In particular, the rectus abdominis muscle flap negates the benefits of a laparoscopic approach and has a high risk of donor-site morbidity. The longer operative time for gluteal turnover flap closure could not be fully attributed to the creation of the flap, because also more extralevator resections were performed in this group. In the pilot study, a median additional operative time of 38 min (IQR 35–44 min) was needed for the gluteal turnover flap [6].

Evaluation of the severity of the specific perineal wound complications revealed that pelvic abscesses were less frequent after gluteal turnover flap closure and there were no cases of fistula, haemorrhage, or necrosis. This is also reflected by the shorter re-admission duration in this patient group. Perineal wound complications after gluteal turnover flap closure seem to be less severe, and probably the perineal wound heals faster compared with primary closure. Due to the retrospective design of this study, it is not possible to precisely identify these healing rates.

It is promising that there were no perineal hernias after gluteal turnover flap closure, despite the larger number of hysterectomies in this group which increases the risk of perineal hernia [13]. The dermis, which is stitched to the contralateral levator remnant, might serve as pelvic floor reconstruction and might therefore lower the risk of perineal hernia after APR. However, this hypothesis needs confirmation in larger studies with longer follow-up.

A major limitation of this study is its retrospective design. Wound complications were scored based on available data in medical records, which made it difficult to evaluate when in time a complication started and ended. Furthermore, no standardised wound scoring was used, causing the scoring to be mainly dependent on subjective interpretation. Since a novel closure technique was performed, perineal wound problems were been monitored very closely afterwards, which may have resulted in an overestimation of wound complications in the gluteal turnover flap group and underestimation in the primary closure group. In addition, the performance of the gluteal turnover flap may seem as a simple surgical procedure. However, a learning curve effect might have negatively influenced the results of the gluteal turnover flap group so far.

## Conclusions

Although the results are promising, outcomes did not reveal statistically significant differences, and a randomised-controlled study is needed to demonstrate its superiority over primary closure regarding perineal wound healing and perineal herniation.

## Data Availability

There was no reproduced material used from other sources in this article.
